# Alterations in Oxidative Stress Markers and Na,K-ATPase Enzyme Properties in Kidney after Fructose Intake and Quercetin Intervention in Rats

**DOI:** 10.3390/life13040931

**Published:** 2023-04-02

**Authors:** Norbert Vrbjar, Jana Vlkovicova, Denisa Snurikova, Barbora Kalocayova, Stefan Zorad, Tijana Culafic, Snezana Tepavcevic, Lubomira Tothova, Dominika Radosinska, Marta Kollarova, Jana Radosinska

**Affiliations:** 1 Centre of Experimental Medicine, Slovak Academy of Sciences, Institute for Heart Research, Dúbravská Cesta 9, 841 04 Bratislava, Slovakia; 2 Institute of Experimental Endocrinology, Biomedical Research Center, Slovak Academy of Sciences, 845 05 Bratislava, Slovakia; 3 Laboratory for Molecular Biology and Endocrinology, “VINČA” Institute of Nuclear Sciences—National Institute of the Republic of Serbia, University of Belgrade, 11000 Belgrade, Serbia; 4 Institute of Molecular Biomedicine, Faculty of Medicine, Comenius University in Bratislava, 811 08 Bratislava, Slovakia; 5 Institute of Medical Biology, Genetics and Clinical Genetics, Faculty of Medicine, Comenius University in Bratislava, Sasinkova 4, 811 08 Bratislava, Slovakia; 6 Institute of Physiology, Faculty of Medicine, Comenius University in Bratislava, Sasinkova 2, 811 08 Bratislava, Slovakia; 7 Premedix Academy, Medená 18, 811 02 Bratislava, Slovakia

**Keywords:** fructose, quercetin, kidney, oxidative stress, Na,K-ATPase, glycemia

## Abstract

The study aimed to characterize the consequences of a 15-week intake of 10% fructose on the kidney, with the focus on oxidative stress markers and properties of the Na,K-ATPase enzyme. Various antioxidants naturally occurring in common food were demonstrated to be protective against fructose-induced deterioration of kidneys. Therefore, we also aimed to observe the effect of 6-week quercetin administration (20 mg/kg/day) that was initiated following the 9-week period of higher fructose intake, by determining the concentration of sodium, potassium, creatinine, urea, and glucose in blood plasma and oxidative status directly in the renal tissue. Kinetic studies of renal Na,K-ATPase were utilized for a deeper insight into the molecular principles of expected changes in this enzyme activity under conditions of presumed fructose-induced renal injury. Fructose intake led to increase in body weight gain, plasma glucose and sodium levels, and deterioration of kidney properties, although some compensatory mechanisms were observable. Quercetin administration improved glycemic control in rats exposed to fructose overload. However, an increase in plasma creatinine, a decrease in GSH/GSSG ratio in renal tissue homogenate, and a controversial effect on renal Na,K-ATPase enzyme suggest that quercetin treatment may not be beneficial in the condition of pre-existing renal pathology.

## 1. Introduction

Excessive consumption of fructose is a hypothesized risk factor contributing to the development of various pathologies such as obesity, diabetes, and hypertension. A diet rich in fructose is followed by a redox imbalance in tissues and organs such as blood, the heart, brain, and kidney [1,2,3,4,5,6]. Data available in databases show that the effect may be time-dependent. Controversial results were observed when experimental animals were fed with a high-fructose diet for a shorter period. A 28-day overload caused fructose-induced hyperglycemia and increased blood serum atherogenicity in rats, but without other manifestations of metabolic syndrome, i.e., dyslipidemia and oxidative stress [7]. In another experiment lasting 30 days, an excessive consumption of fructose led to increased lipid peroxidation and impaired antioxidant status in the kidneys of rats [8]. When rats were exposed to excess fructose for 35 days, oxidative stress was documented by an increased malondialdehyde presence in the blood plasma, while their kidneys showed only a slight, statistically insignificant, decrease in the activity of the antioxidant enzymes catalase and glutathione peroxidase [9]. Rats maintained on a high-fructose diet for 60 days showed increased levels of peroxidation end-products, decreased antioxidant status, and increased protein damage and lipid accumulation in kidney tissue [10].

In studies focused on the correct condition of the kidneys, renal Na,K-ATPase activity represents a very important indicator. This enzyme, in its key role in maintaining the correct sodium ion concentration in kidney cells, as well as in the regulation of the overall sodium homeostasis in the organism, is highly sensitive to the oxidative state of the kidney tissue in various pathologic situations [11,12,13,14]. Despite intensive studies of the effect of excessive fructose intake on renal functions, relatively few studies have addressed the properties of renal Na,K-ATPase under these conditions. In addition, the previously published data are controversial. Diet enriched in 20% fructose causing salt-sensitive hypertension did not induce alteration in the expression of Na,K-ATPase in kidneys [15]. On the other side, in rats drinking solution of fructose (10% *w*/*v*) causing an imbalance between the renin–angiotensin system (RAS) and the renal dopaminergic system, as well as insulin resistance, the symptoms were accompanied by time-dependent increase in activity of renal tubular Na,K-ATPase in the 4–12-week period of fructose overload. The stimulatory effect of fructose on the activity of Na,K-ATPase was prevented by pharmacological inhibition of RAS by losartan [5]. A protective effect against fructose-induced deterioration of the oxidative state in the kidneys was also demonstrated after the administration of various antioxidants occurring in common food [7,8,9,10,16,17,18]. Among such natural antioxidants, quercetin was identified as a promising “renoprotective” agent [19]. Although the safety of quercetin was proved by multiple studies, there are also indices regarding its potential prooxidative properties [20,21].

Considering the known facts, the aim of this study was to provide detailed characteristics of kidney status after chronic fructose overload, as well as the effect of quercetin administration in rats, by determining the concentration of sodium, potassium, creatinine, urea, and glucose in blood plasma and oxidative status directly in the renal tissue. For a deeper insight into the molecular principles of expected changes in the renal Na,K-ATPase activity under conditions of the expected fructose-induced renal injury, kinetic studies of this enzyme were utilized. In addition, this study was designed to reveal the effect of treatment in the case of already-impaired kidney status. Thus, quercetin was administered after a period during which the rats were exposed to higher fructose intake.

## 2. Materials and Methods

### 2.1. Experimental Model

The experimental protocol was approved in accordance with the EU Directive (2010/63/EU) by the Ethical Committee for Care and Use of Laboratory Animals at the Vinča Institute of Nuclear Sciences, National Institute of the Republic of Serbia, University of Belgrade.

Three-week-old male Wistar rats (n = 48) were weaned from their mothers and placed three per cage to avoid placing animals from the same litter in the same cage. For the first 9 weeks of the experiment, the rats were initially divided into 2 groups of 24 animals each. One group drank tap water and the second group drank 10% fructose solution. Fructose was purchased from Omnia Nisasta Sanayi ve Ticaret A.S. (Adana, Turkey). Both groups were allowed to drink their beverages ad libitum, as well as having free access to pelleted food (Veterinary Institute, Subotica, Serbia). The dose and duration of fructose intake were based on previous observations that rats developed insulin resistance under such conditions [22].

Following the first part of the experiment, both groups were divided into two subgroups. Two of the subgroups received quercetin by gastric gavage (20 mg/kg/day, Sigma-Aldrich, cat. number Q4951, St. Louis, MO, USA) for an additional 6 weeks. The respective control groups were administered by gavage with a solution of 1% methylcellulose (Sigma-Aldrich, M 6385 from) in distilled water serving as a vehicle for quercetin.

Designation of experimental groups:

C: control—standard food and tap water throughout both periods of the experiment (9 + 6 weeks), and administration of vehicle—1% methylcellulose, by gavage for the last 6 weeks.

Q: quercetin-treated—standard food and tap water throughout both periods of the experiment (9 + 6 weeks), and administration of quercetin in 1% methylcellulose by gavage for the last 6 weeks.

F: standard food and 10% fructose in tap water throughout both periods of the experiment (9 + 6 weeks), and administration of vehicle—1% methylcellulose, by gavage for the last 6 weeks.

FQ: standard food and 10% fructose in tap water throughout both periods of the experiment (9 + 6 weeks), and administration of quercetin in 1% methylcellulose by gavage for the last 6 weeks.

**Figure 1:**
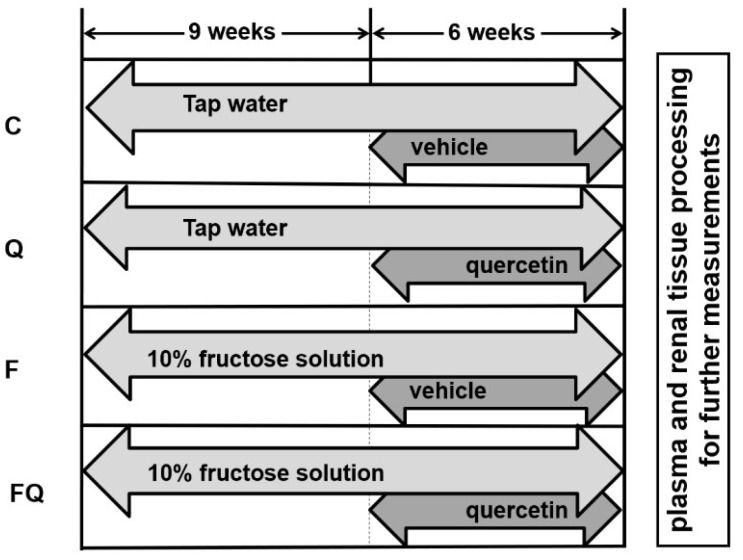
Experimental design. Abbreviations: C—control rats with standard diet, Q—rats with standard diet for 15 weeks and after administration of quercetin for the last 6 weeks of the experiment, F—rats fed by fructose for 15 weeks, FQ—rats fed by fructose for 15 weeks and after administration of quercetin for the last 6 weeks of the experiment.

The overall experimental design is available in Figure 1.

The animals were housed in a temperature-controlled room (22 ± 1 °C) under a light:dark regime of 12:12, with light on from 7.00 a.m. At the end of the experiment, the experimental animals were sacrificed by decapitation without anesthesia, thus avoiding possible side effects that could affect some monitored parameters (which are part of another study). Rats fasted overnight before the sacrifice.

Blood samples were collected in commercially available heparinized tubes (Becton, Dickinson and Company, Plymouth, UK). Blood plasma was isolated by centrifugation at 1000× *g* for 10 min.

### 2.2. Biochemical Analyses

The concentration of sodium, potassium, creatinine, urea, and glucose in the blood plasma was measured using a Vitros 250 autoanalyzer (Johnson&Johnson, Rochester, NY, USA).

### 2.3. Markers of Oxidative Stress, Carbonyl Stress, and Antioxidant Status

Markers of oxidative damage (lipid peroxidation and protein oxidation), carbonyl stress, and antioxidant status were assessed in the kidney tissue that was homogenized in phosphate-buffered saline (PBS, pH = 7.2) to prepare 10% homogenates. Afterwards, the samples were centrifuged at 4000× *g* for 10 min. The supernatant was collected and stored at −20 °C until further analyses. Measurements (spectrophotometric and fluorescent) were performed by using a Synergy H1 Hybrid Multi-mode Reader (Agilent, Santa Clara, CA, USA), and all chemicals used in the analysis were purchased from Sigma-Aldrich (Bratislava, Slovakia). We proceeded according to previous studies [23,24].

Lipid peroxidation was estimated by measuring thiobarbituric acid reactive substances (TBARS). Briefly, 20 µL of samples and standards (1,1,3,3-tetraethoxypropane) were mixed with 30 µL of distilled water, 20 µL of 0.67% thiobarbituric acid, and 20 µL of glacial acetic acid. The microplates were shortly mixed and incubated at 95 °C for 45 min. Subsequently, the microplates were left to cool at room temperature and then 100 µL of n-butanol was added into the microplates and centrifuged (2000× *g*, 10 min, 4 °C). A total of 70 µL of the upper phase was transferred into a new dark microplate and fluorescence was measured at λ_ex_ = 515 nm and λ_em_ = 553 nm.

By measuring advanced oxidation protein products (AOPP), the marker of protein oxidative damage could be determined. For the analysis, 200 µL of samples and standards (chloramine T mixed with potassium iodide) were mixed with 20 µL of glacial acetic acid. After mixing for 2 min, absorbance was measured at λ = 340 nm.

Regarding the marker of carbonyl stress, advanced glycation end-products (AGEs) were determined. Then, 20 µL of samples were mixed with 180 µL of PBS in the dark microtiter plate and autofluorescence of the samples was measured at λ_ex_ = 370 nm and λ_em_ = 440 nm. Standards (AGE-BSA) were added (mixed with 180 µL PBS) and fluorescence was measured at λ_ex_ = 370 nm and λ_em_ = 440 nm again.

Fructosamine (FRUC) was used as a marker of advanced glycation of proteins. Then, 20 µL of samples and standards (16 mmol/L 1-deoxy-morpholino-d-fructose) were mixed with 100 µL of 0.25 mmol/L nitro blue tetrazolium, containing 0.1 mol/L sodium carbonate buffer (pH 10.35) and 1 mmol/L nitro blue tetrazolium. Then, samples were incubated at 37 °C for 15 min and absorbance was measured at λ = 530 nm.

For the measurement of total antioxidant capacity (TAC), 20 µL of samples and standards (1 mmol/L TROLOX mixed with dimethylsulfoxide and distilled water) were mixed with 200 µL of acetate solution (0.4 mol/L CH_3_COONa reagent and 0.4 mol/L glacial acetic acid, pH 5.8). The absorbance was measured at λ = 660 nm as blank. Then, 20 µL of ABTS solution (2.2′-azino-bis(3-ethylbenzothiazoline-6-sulfonic acid) with acetate buffer) was added, and, after 5 min of gentle mixing, the absorbance was measured at λ = 660 nm again.

Ferric reducing antioxidant power (FRAP) was used as a marker of antioxidant status. A 200 µL measure of warmed (37 °C) FRAP reagent (containing 3 mol/L acetate buffer, pH 3.6, 10 mmol/L tripyridyl-s-triazine, 20 mmol/L FeCl_3_·6H_2_O, and water) was prepared fresh and pipetted into a microplate. Absorbance of FRAP reagent was measured at λ = 593 nm as a blank. Afterward, 20 µL of samples and standards (100 mmol/L FeSO_4_·7H_2_O) were added and gently vortexed for 4 min. The absorbance was measured at λ = 593 nm.

The ratio of reduced and oxidized glutathione (GSH/GSSG) is used as a general marker of oxidative stress. GSH was measured by mixing 10 µL of samples and standards (1 mmol/L L-glutathione reduced) with 10 µL of O-phtalaldehyde solution (1 mg/mL) and 180 µL of the PBS (100 mmol/L with 2.5 mM EDTA-Na_2_). Afterward, the mixture was incubated for 15 min at room temperature. Fluorescence was measured at λ_ex_ = 350 nm and λ_em_ = 460 nm. GSSG was measured by mixing 25 µL of samples and standards ((-)-glutathione, oxidized) with 10 µL of N-ethylmaleimide (5 mg/mL). After incubation (for 40 min at room temperature), 10 µL of the mixture was transferred into the new dark microtiter plate with addition of 10 µL O-phtalaldehyde solution (1 mg/mL) and 180 µL NaOH (0.1 mmol/L). After 15 min of incubation and gentle vortexing at room temperature, fluorescence was measured at λ_ex_ = 350 nm and λ_em_ = 460 nm.

### 2.4. Na,K-ATPase-Enzyme Kinetic Measurements

The isolation of plasma membrane fractions from kidney tissues, determination of protein concentration in samples, and kinetic measurements of Na,K-ATPase enzyme were performed as described previously [23,25].

All measurements were performed at 37 °C with the application of 10 µg/mL membrane protein. Na,K-ATPase activity was measured using a buffer comprising (in mmol/L) 4 MgCl_2_, 100 NaCl, 10 KCl, and 50 TRIS (pH = 7.4). Following the 20-minute preincubation in a substrate-free medium, ATP in the range of 0.16–8.00 mmol/L was added to initiate the enzyme reaction that was stopped after 20-min incubation by addition of 12% ice-cold trichloroacetic acid. To establish the Na,K-ATPase activity, ATP hydrolysis occurring in the presence of Mg^2+^ ions was subtracted. The same approach was applied to determine the Na,K-ATPase enzyme kinetics for sodium activation. In this case, the amount of ATP was constant (8 mmol/L), while the NaCl concentration varied in the range of 2–100 mmol/L.

### 2.5. Na,K-ATPase-Electrophoresis and Immunochemical Western Blot Analysis

Homogenized renal tissue was provided for particular fraction preparation by 3-step centrifugation as previously described [13]. After electrophoretic separation of proteins (20 µg per each lane) in 12% sodium dodecyl sulfate-polyacrylamide gel, the samples were transferred to a nitrocellulose membrane. The membrane was incubated overnight with primary antibodies against individual subunits of Na,K-ATPase (α1, 1:250, A-277, Sigma-Aldrich, St. Louis, MO, USA; β1, 1:200, sc-21713, Santa Cruz Biotechnology, Dallas, TX, USA), and subsequently incubated for 1.5 h with a horseradish peroxidase-linked secondary anti-mouse antibody (1:1000, #7076C, Cell Signaling Technology, Denver, CO, USA). For protein visualization, an enhanced luminol-based chemiluminescence was applied. The quantification of the relevant bands was assessed densitometrically using Image J software, and subsequently normalized to β-actin (1:1000, ab6276, Abcam, Cambridge, UK) serving as a loading control [13].

### 2.6. Statistical Analysis

Data are presented as means ± standard deviations (SD). Data normality was analyzed by the D’Agostino–Pearson test. The Grubbs test was used to identify outliers objectively. For enzyme kinetic parameters, namely, maximal velocity of enzyme reaction (V_max_), and the Michaelis–Menten constant, i.e., the concentration of substrate (or cofactor) necessary for half-maximal velocity of enzyme reaction, direct non-linear regression of the obtained data was used. To determine the effects of fructose intake, as well as quercetin treatment, two-way analysis of variance (ANOVA) was applied. Afterwards, Tukey’s multiple-comparisons test was used to reveal differences between individual experimental groups. They were considered significant when the *p*-value was lower than 0.05. GraphPad Prism 7.02 and SigmaPlot 13 software were used for statistical analyses.

## 3. Results

### 3.1. Basic Characteristics of Experimental Animals

After random selection of all animals at the beginning of the experiment, their body weight was approximately 53 g, with no statistical differences among experimental groups. At the end of the experiment, two-way ANOVA revealed that fructose intake resulted in a statistically significant increase in body weight in rats, independent of quercetin treatment (F_(1,44)_ = 9.089, *p* = 0.004).

The rats from the F group had lower food intake, but higher liquid (i.e., 10% fructose) and energy intake, in comparison with the rats from the C group (*p* < 0.0001 for each comparison). Quercetin treatment attenuated these changes—it resulted in an increase in food intake (*p* = 0.0009), and decrease in liquid (*p* < 0.0001) as well as energy intake (*p* = 0.035) in animals that drank 10% fructose (F vs. FQ group).

Regarding the weight of left kidneys used for all investigations, its absolute value was higher after fructose intake (F_(1,44)_ = 4.226, *p* = 0.046). However, normalized kidney weight, i.e., adjusted to body weight, was lower in quercetin-treated rats, independent of fructose intake (F_(1,44)_ = 6.517, *p* = 0.014). Data regarding all determined parameters with statistical analysis are available in Table 1.

### 3.2. Biochemical Analysis of Blood Plasma

Excessive consumption of fructose in the drink alone caused an 11% increase in glucose levels (C vs. F group, *p* = 0.01). Administration of quercetin ameliorated this negative effect of fructose intake to a level even slightly lower compared with control rats (F vs. FQ group, *p* < 0.0001). The creatinine content was significantly higher after administration of quercetin in fructose-fed rats (F vs. FQ group, *p* < 0.05). According to the two-way ANOVA, the fructose intake led to an increase in plasma urea level (F_(1,43)_ = 8.95, *p* = 0.005), however, without significant differences among groups in multiple comparison test.

Concentration of sodium ions was significantly higher in rats with excessive intake of fructose, independent of the presence or absence of quercetin (F_(1,42)_ = 13.74, *p* < 0.001). Concentration of potassium ions in blood plasma showed significantly lower values by 15–17% in rats with excessive consumption of fructose, independent of quercetin administration (F_(1,42)_ = 43.27, *p* < 0.0001).

All data with statistical analysis are available in Table 2.

### 3.3. Markers of Antioxidant Status and Oxidative and Carbonyl Stress in the Renal Tissue

Regarding the marker of lipid peroxidation, TBARS, the concentration was higher in rats fed with fructose (F_(1,43)_ = 12.66; *p* = 0.0009), independent of quercetin administration, with a significant difference between the C and F groups (*p* = 0.01) in the multiple-comparison test. An administration of quercetin decreased AOPP concentrations, independent of fructose intake (F_(1,41)_ = 33.7; *p* < 0.0001). AOPP levels were lower in the Q group in comparison with the C group (*p* = 0.0002), and lower in the FQ group in comparison with the F group (*p* = 0.0052). Carbonyl stress estimated by the determination of AGEs was increased after quercetin administration (F_(1,42)_ = 4.875; *p* = 0.033), however, determination of the FRUC concentration revealed the opposite—FRUC was lower in quercetin-treated rats, independent of fructose intake (F_(1,36)_ = 16.39; *p* = 0.0003). In multiple comparisons, FRUC concentration was lower in the FQ group in comparison with the F group (*p* = 0.0124).

When focusing on antioxidant status markers, TAC was higher in rats after fructose intake, independent of quercetin administration (F_(1,43)_ = 10.57; *p* = 0.0022), but without significant differences in the multiple-comparison test. FRAP was affected by quercetin treatment in a different way depending on the fructose intake (interaction between factors: F_(1,42)_ = 11; *p* = 0.0019). In comparison with the C group, FRAP was higher in the Q group (*p* = 0.0094). Interestingly, FRAP was also higher in the F group when compared with the C group (*p* = 0.0033).

A general indicator of the redox state, GSH/GSSG ratio, was lowered after the quercetin treatment (F_(1,43)_ = 18.05; *p* = 0.0001), as well as after the fructose intake (F_(1,43)_ = 23.02; *p* < 0.0001). Additionally, in comparison with rats assigned to the C group, GSH/GSSG ratio was lower in those rats in the Q group (*p* = 0.0092) along with the F group (*p* = 0.0031) relative to the C group. The difference between the F and the FQ groups with a *p*-value of 0.05 was noted as close to statistical significance. The summary of obtained data with statistical analysis is available in Table 3.

### 3.4. Renal Na,K-ATPase Enzyme Characterization of Kinetic Parameters

**Figure 2:**
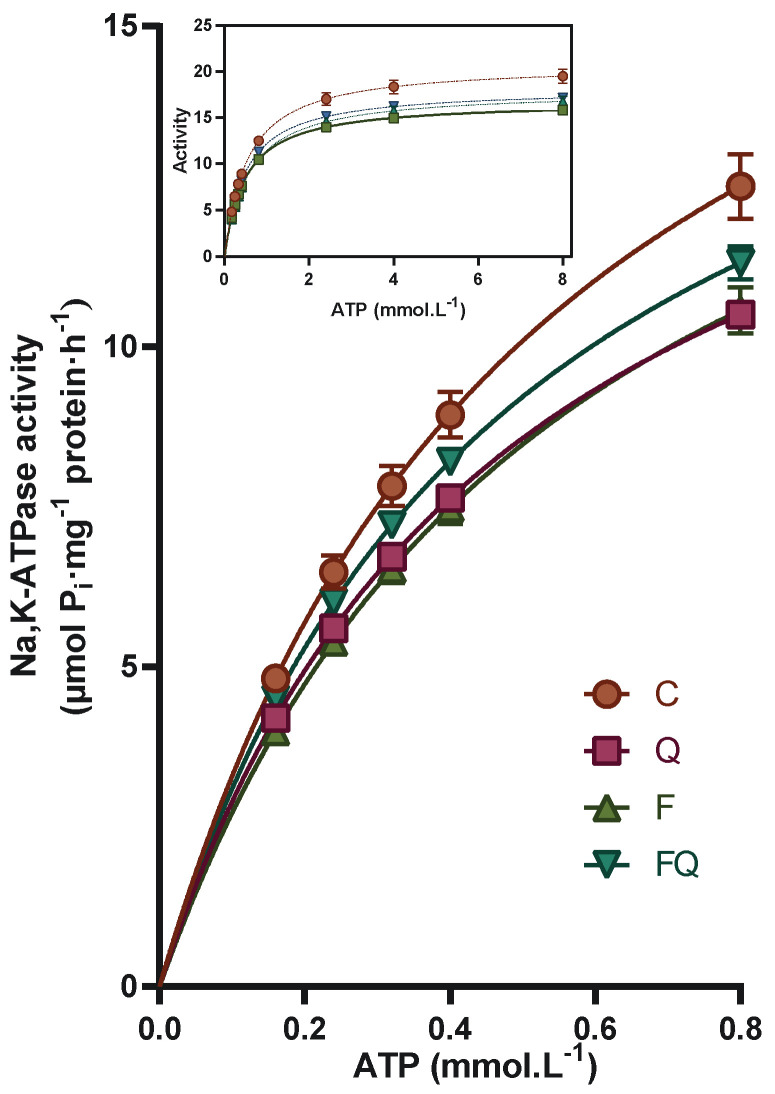
Na,K-ATPase enzyme activity as a function of increasing concentration of ATP substrate. Abbreviations: C—control rats with standard diet, Q—rats with standard diet for 15 weeks and after administration of quercetin for the last 6 weeks of the experiment, F—rats fed by fructose for 15 weeks, FQ—rats fed by fructose for 15 weeks and after administration of quercetin for the last 6 weeks of the experiment.

Enzyme activation with increasing concentration of its energy substrate showed reduced activity values in all experimental groups compared with the control group throughout the applied ATP concentration range (Figure 2). Fructose alone caused a stable decrease in enzyme activity by about 15% when comparing the F and C groups. Comparison of the FQ group with the F group showed a slight increase in the Na,K-ATPase activity, especially in the presence of lower concentrations of substrate, representing a 13% increase in the presence of 0.16 mmol/L ATP. Quercetin administration to control rats induced by itself inhibition of the enzyme ranging from 13% to 19% gradually with increasing concentration of ATP (Figure 2).

**Figure 3:**
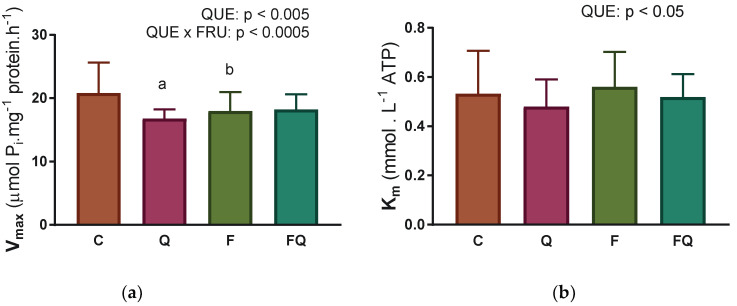
Kinetic parameters of Na,K-ATPase enzyme during its activation with an increasing concentration of ATP substrate: (**a**) Maximal velocity of enzyme reaction—V_max_; (**b**) Concentration of ATP substrate that is necessary to half-maximal velocity of enzyme reaction—K_m_. Abbreviations: C—control rats with standard diet, Q—rats with standard diet for 15 weeks and after administration of quercetin for the last 6 weeks of the experiment, F—rats fed by fructose for 15 weeks, FQ—rats fed by fructose for 15 weeks and after administration of quercetin for the last 6 weeks of the experiment, QUE—effect of quercetin administration, FRU—effect of fructose intake. Statistical significance—a: *p* < 0.0001 vs. C; b: *p* < 0.01 vs. C.

Evaluation of kinetic parameters according to the Michaelis–Menten equation showed a decrease in the V_max_ value in all experimental groups by 13–20% when compared with control group (Figure 3a). The values of the second kinetic parameter K_m_ were significantly reduced as a result of quercetin treatment, by 10–13%, independent of fructose consumption (Figure 3b).

**Figure 4:**
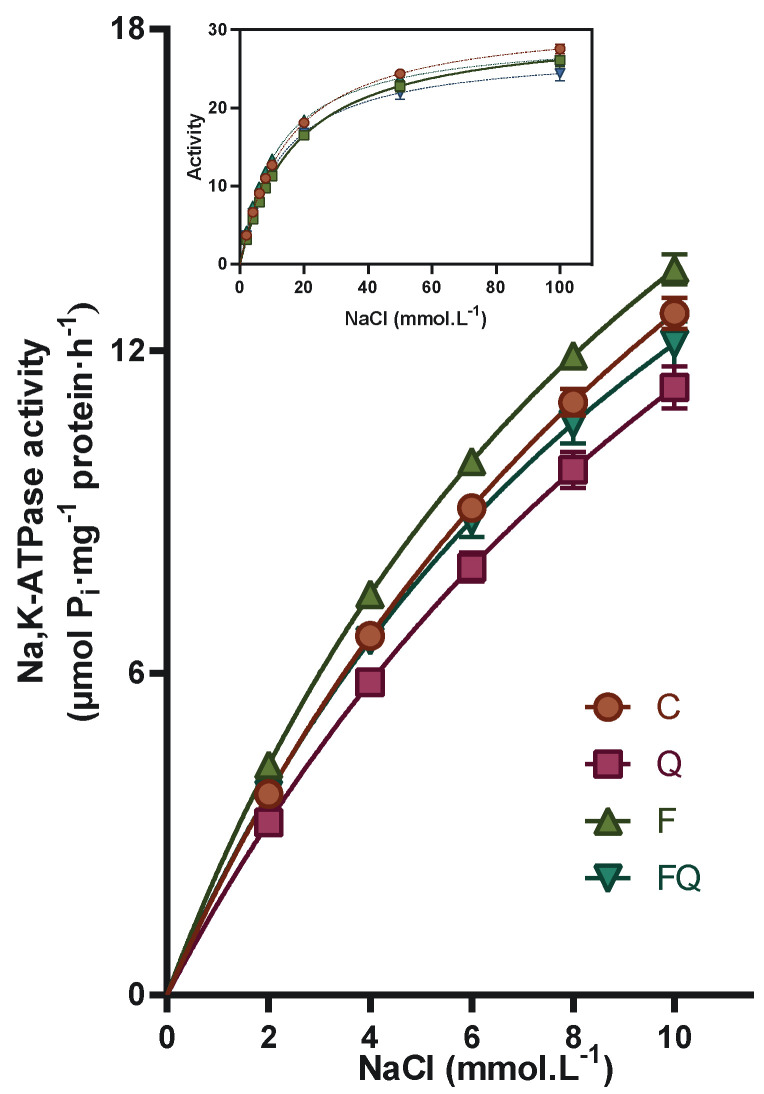
Na,K-ATPase enzyme activation in NaCl concentrations ranging from 2 to 10 mmol/L. Inset—Na,K-ATPase enzyme activation in the whole investigated concentration range of NaCl in the kidney tissue. Abbreviations: C—control rats with standard diet, Q—rats with standard diet for 15 weeks and after administration of quercetin for the last 6 weeks of the experiment, F—rats fed by fructose for 15 weeks, FQ—rats fed by fructose for 15 weeks and after administration of quercetin for the last 6 weeks of the experiment.

When activating the enzyme with an increasing concentration of sodium ions, the activities in the fructose group (F) were higher by more than 10% in the NaCl range below 10 mmol/L (Figure 4). On the other hand, in the presence of a higher concentration of the aforementioned ion above 20 mmol/L, the enzyme activity was lower by 5% compared to the control group. Administration of quercetin to fructose-fed rats (FO group) produced a 9–12% decrease in enzyme activity over the entire examined NaCl range when compared with the F group. Administration of quercetin alone to rats drinking tap water produced a reduction in Na,K-ATPase activity, with the highest effect reaching a 14% decrease at 2 mmol/L NaCl. In the presence of a higher concentration of NaCl, the inhibitory effect of quercetin gradually decreased to 5% of that observed in the presence of 100 mmol/L NaCl.

**Figure 5:**
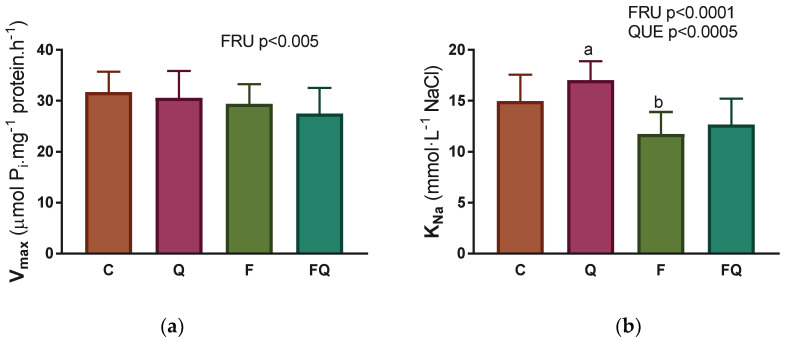
Kinetic parameters of Na,K-ATPase enzyme during its activation with an increasing concentration of Na^+^ ions: (**a**) Maximal velocity of enzyme reaction—V_max_; (**b**) Concentration of Na^+^ necessary to half-maximal velocity of enzyme reaction—K_Na_. Abbreviations: C—control rats with standard diet, Q—rats with standard diet for 15 weeks and after administration of quercetin for the last 6 weeks of the experiment, F—rats fed by fructose for 15 weeks, FQ—rats fed by fructose for 15 weeks and after administration of quercetin for the last 6 weeks of the experiment, QUE—effect of quercetin administration, FRU—effect of fructose intake. Statistical significance—a: *p* < 0.0001 vs. C; b: *p* < 0.01 vs. C.

Regarding the kinetic parameters, the V_max_ value was reduced in rats with 10% fructose intake, independent of quercetin treatment (Figure 5a). The K_Na_ values were lowered in both groups drinking high fructose beverage by 21–25%, independent of quercetin treatment (Figure 5b). In the group of rats treated with quercetin and maintained on a standard diet, the value of V_max_ remained unaffected, but the value of K_Na_ was significantly increased by 14% (C vs. Q group, *p* = 0.005).

### 3.5. Renal Na,K-ATPase Enzyme-Subunit Expressions

**Figure 6:**
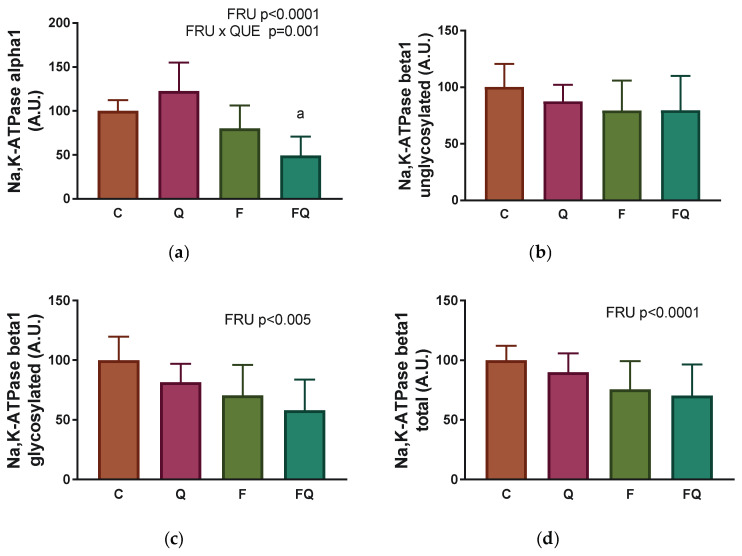
Renal Na,K-ATPase enzyme—a protein expression of individual subunits: (**a**) Alpha1; (**b**) Beta1 unglycosylated; (**c**) Beta1 glycosylated; (**d**) Beta1 total. Abbreviations: C—control rats with standard diet, Q—rats with standard diet for 15 weeks and after administration of quercetin for the last 6 weeks of the experiment, F—rats fed by fructose for 15 weeks, FQ—rats fed by fructose for 15 weeks and after administration of quercetin for the last 6 weeks of the experiment, QUE—effect of quercetin administration, FRU—effect of fructose intake. Statistical significance—a: *p* < 0.05 vs. F.

Expression of the catalytic α1 subunit of Na,K-ATPase was significantly reduced in rats consuming 10% solution of fructose instead of drinking water, independent of quercetin administration (F_(1,38)_ = 38.18, *p* < 0.0001). Administration of quercetin induced fructose dependent effects on the protein expression of the α1 subunit (interaction between fructose intake and quercetin treatment: (F_(1,38)_ = 12.6, *p* = 0.001), while the expression was higher in the F group when compared with the FQ group (*p* = 0.0311) (Figure 6a). The presence of the unglycosylated form of the β1 subunit was not affected on a statistically significant level among experimental groups (Figure 6b). However, the level of the glycosylated β1 subunit, and subsequently total level β1 subunit of Na,K-ATPase, was lower in rats consuming fructose as compared with rats drinking tap water, independent of quercetin treatment (F_(1,24)_ = 9.847, *p* = 0.0045 for glycosylated form; F_(1,24)_ = 8.325, *p* = 0.0081 for total form), without significant differences in multiple comparison test (Figure 6c,d).

## 4. Discussion

This study was focused on the consequences of higher fructose intake on kidney tissue with an emphasis on Na,K-ATPase enzyme properties, along with investigating the effect of quercetin intervention. The decision to test the effect of quercetin administration was based on our previous observations of its potential benefits but also controversial effects in different pathophysiological conditions [26,27,28,29].

Previous studies showed that drinking 20% fructose solution for six or twelve weeks induced approximately 50% body weight gain as compared with control rats [30,31]. During the present study, drinking of 10% fructose solution for 15 weeks induced an 8% increase in body weight as compared with rats drinking tap water. This finding corresponds to a moderate increase in body weight of rats drinking 10% solution of fructose for 14 weeks [5]. However, a longer, 36-week period of drinking a 10% fructose solution induced a 33% increase in body weight [32]. Therefore, the body weight gain induced by excessive consumption of fructose in the drink of laboratory animals depends on the concentration of the fructose, as well as the time of exposure. The increase in body weight observed in the F group in this study is in line with higher energy intake that can be ascribed to an increase in liquid (i.e., 10% fructose) drinking as the food intake was lowered. Interestingly, an administration of quercetin to rats with 10% fructose intake induced an increase in energy intake from pelleted food, while the desire to drink fructose solution was lowered.

It is widely accepted that, besides promoting obesity, higher consumption of fructose is accompanied by elevated blood glucose levels. Our results showed that having already consumed 10% fructose increased the glucose level significantly by 11%. This finding is consistent with previous observations documenting continuously elevated glycemia in rats exposed to 14 weeks of excessive fructose consumption [5,33]. The dose dependency of the above fructose-induced effect is supported by a study documenting a significant increase in glucose level by 27% in rats consuming a 20% fructose solution [30]. Serious metabolic complications were also documented in young human volunteers depending on the fructose content in beverages already after 2-week consumption of 10–25% fructose solution [34,35]. In fructose-induced obesity, eventually leading to the development of all components of metabolic syndrome, elevated uric acid level was shown to be an important causal factor [36,37]. In our experimental settings, quercetin administration exerted a clear glycemia-lowering effect in rats exposed to fructose intake. However, contradictory findings were observed regarding the effect of quercetin on glycemia in human studies [38]. Similarly, quercetin administration led to an unexpected increase in plasma creatinine levels in rats receiving the fructose instead of drinking water, even though the vast majority of animals had plasma creatinine and urea concentrations in the range of normal values [39].

The slight but statistically significant increase in plasma sodium concentration may indicate a predisposition to sodium retention in the organism as a consequence of higher fructose intake. It was already suggested that excessive consumption of fructose contributes to increased salt retention in the organism [33,40,41].

Therefore, another important objective of this study was to determine whether excessive consumption of fructose in rats is followed by functional changes in the Na,K-ATPase enzyme in the kidney. The enzyme kinetic studies applied in the present study provide deeper insight into the energy supply via hydrolysis of ATP and affinity changes of the Na-binding site in the Na,K-ATPase molecule. It was recognized that Na,K-ATPase converts a large proportion of the intracellular ATP production to active Na^+^, K^+^ transport. In the kidney, approximately 90% of oxygen extracted by mitochondria is used for the work required for Na^+^ reabsorption in the nephron [42]. Thus, energy utilization by Na,K-ATPase investigated during activation of the enzyme with increasing concentrations of the substrate ATP is a very important factor for characterizing the enzyme functionality. The reduced enzyme activities observed upon stimulation of Na,K-ATPase with ATP suggest a reduced utilization of the energy substrate after excessive fructose consumption. The observed impairment of energy utilization can be attributed to a decrease in the number of active Na,K-ATPase molecules, as indicated by decreased V_max_ value. This suggestion is also supported by lowered protein expression of the catalytic α1 subunit that contains the binding sites for ATP, as well as for sodium ions, in fructose-fed animals. Normotensive Wistar rats were used in this study. Different results, i.e., an increase in protein expression of the α1 subunit of Na,K-ATPase, was observed in spontaneously hypertensive rats following 7-week fructose feeding [40]. In addition, Na,K-ATPase seems to be embedded less optimally into the cell membrane in our rats after fructose intake. The β1 subunit functions as the chaperon for the correct insertion of the protein molecule. The decreased presence of the glycosylated form of the β1 subunit may be of crucial importance, as the glycosylation is needed in the process of optimal enzyme implementation into the cell surface membrane as documented previously [43]. Similar alterations in Na,K-ATPase activity were documented in several studies of experimental diabetes in rats [23,44,45,46]. However, overconsumption of fructose by itself did not affect the ATP-binding area of the enzyme, as shown by the stable value of K_m_.

Administration of quercetin to control animals produced an even more pronounced effect compared with the effect induced by fructose, as evidenced by the lowest V_max_ value among all four investigated groups of animals. This finding differs from data regarding the higher expression of the α1 subunit in rats subjected to quercetin solely. This discrepancy between kinetic and protein expression data of the Na,K-ATPase enzyme may indicate that quercetin promotes the synthesis of α1 molecules. However, they are inactive, probably due to post-translational changes of the enzyme molecule. This negative effect was at least partially compensated by the improved enzyme ability to bind ATP, as indicated by a lowered K_m_ value after quercetin administration, independent of fructose intake. Taken together, both fructose intake and quercetin administration affect the utilization of ATP energy substrate by the Na,K-ATPase enzyme in kidneys by different mechanisms.

Regarding sodium-binding properties, excessive fructose consumption improved the ability of renal Na,K ATPase to bind sodium, as shown by a significant (25%) decrease in the K_Na_ value in comparison with the control group. Improved sodium binding resulted in the highest effect on the enzyme in the presence of physiologically relevant sodium concentrations, i.e., lower than 20 mmol/L, corresponding to intracellular conditions. The observed increase in sodium binding can at least partially contribute to the increased sodium level in blood plasma after overconsumption of fructose. In contrary to fructose intake, administration of quercetin to control animals deteriorated the sodium binding ability of the enzyme, as indicated by a significant increase in K_Na_ value. The unchanged level of sodium in blood plasma of quercetin-treated rats was probably balanced by an improved ability of the Na,K-ATPase in binding the energy substrate ATP as shown during activation of the enzyme with ATP.

Markers of oxidative and carbonyl stress, as well as antioxidant state, were determined in order to characterize the condition of the kidney upon fructose intake, since an excessive fructose intake can lead to oxidative stress in the kidney as documented previously [47]. Consistently with this observation, a negative effect of drinking 10% fructose on the kidney was documented in this study, specifically in terms of an increase in lipid peroxidation, and decrease in thiol-redox balance estimated by the determining the GSH/GSSG ratio. Interestingly, the increased kidney total antioxidant capacity found in our experiment might suggest a response of antioxidant defenses to an increase in tissue oxidative stress [48]. Determination of oxidative stress markers in kidney tissue was also important in rats after administration of lipid-soluble antioxidant quercetin. Antioxidant action of quercetin was well-documented in terms of a decrease in markers of protein oxidation, as well as fructosamine concentration. However, other markers of carbonyl stress—concentration of AGEs, and especially the GSH/GSSG ratio—showed the opposite trend. Therefore, a clear antioxidant action of quercetin without any undesirable consequences in the renal tissue of our experimental animals was not confirmed by our study. The decrease in GSH concentration following the quercetin treatment was previously observed in a rat lung epithelial [49] and neural cell line [50]. This may indicate that quercetin, being a strong antioxidant, can induce an imbalance in cellular antioxidant defense mechanisms via formation of thiol reactive metabolites [49]. This finding is in line with the previous observation of potential prooxidative properties of quercetin depending on the dosage and the time of exposure. Two weeks of daily administration of quercetin in three various doses (10, 50, 100 mg/kg BW) to rats with doxorubicin-induced nephrotoxicity exerted a protective effect in the case of the lowest dose only. Administration of higher doses induced deleterious effects in the kidney [20]. Additionally, quercetin (70 mg/kg diet) aggravated the kidney status in a chronic 28-week model of streptozotocin-induced diabetes in rats [21]. What can also modify the action of quercetin, and should be taken into the consideration, is the presence of pre-existing pathology. In this study, the quercetin was administered to rats previously subjected to higher fructose intake, i.e., in the condition when renal functions could be already impaired. In the case of simultaneous deterioration and quercetin treatment, the overall effect can be different. Nevertheless, whether the quercetin is potentially harmful only when administered in higher doses should be investigated in further studies, as our dose, 20 mg/kg of quercetin per day, is generally not considered high.

## 5. Conclusions

Summarizing the results of this study, 10% fructose intake lasting 15 weeks led to signs of metabolic syndrome (increased BW gain and blood glucose level), and deterioration of renal properties, although some compensatory mechanisms were observable as well. The treatment of quercetin that was initiated following the 9-week exposure of rats to fructose overload was clearly beneficial regarding the glycemic control. Nevertheless, quercetin action was not always protective when focusing on kidney status. An increase in plasma creatinine, decrease in GSH/GSSG ratio in renal tissue homogenate, and controversial effect on renal Na,K-ATPase enzyme should be taken into consideration in recommendations of quercetin treatment in the condition of pre-existing renal pathology.

## Figures and Tables

**Table 1:** Basic characteristics of experimental animals.

Parameter	Experimental Groups				Two-Way ANOVA		
	C (n = 12)	Q (n = 12)	F (n = 12)	FQ (n = 12)	QUE	FRU	Int.
BW start (g)	54 ± 3	53 ± 5	53 ± 3	53 ± 4			
BW end (g)	535 ± 37	539 ± 52	577 ± 42	572 ± 39		**	
BW gain (g)	481 ± 36	486 ± 50	524 ± 42	519 ± 40		**	
Food intake (g/rat/day)	24.5 ± 0.7	24.0 ± 0.8	17.8 ± 1.0 ^a^	19.3 ± 1.1 ^b^	*	****	***
Liquid intake (mL/rat/day)	61.0 ± 3.9	58.8 ± 7.3	81.8 ± 9.5 ^a^	58.1 ± 12.5 ^c^	****	***	***
Energy intake (kJ/rat/day)	269 ± 8	264 ± 9	336 ± 27 ^a^	312 ± 30 ^d^	*	****	
KW (g)	1.76 ± 0.16	1.67 ± 0.13	1.82 ± 0.18	1.78 ± 0.13		*	
KW/BW (mg/g)	3.29 ± 0.23	3.10 ± 0.19	3.23 ± 0.17	3.12 ± 0.19	*		

Abbreviations: C—control rats with standard diet, Q—rats with standard diet for 15 weeks and after administration of quercetin for the last 6 weeks of the experiment, F—rats fed by fructose for 15 weeks, FQ—rats fed by fructose for 15 weeks and after administration of quercetin for the last 6 weeks of the experiment, QUE—effect of quercetin administration, FRU—effect of fructose intake, Int.—interaction between the quercetin administration and fructose intake, BW—body weight, KW—kidney weight. Data represent mean ± SD. Statistical significance—a: *p* < 0.0001 vs. C; b: *p* < 0.001 vs. F, c: *p* < 0.0001 vs. F; d: *p* < 0.05 vs. F; * *p* < 0.05, ** *p* < 0.01, *** *p* < 0.001, **** *p* < 0.0001 for the corresponding factors and their interaction.

**Table 2:** Selected biochemical parameters measured in blood plasma.

Parameter	Experimental Groups				Two-Way ANOVA		
	C (n = 12)	Q (n = 11)	F (n = 11)	FQ (n = 12)	QUE	FRU	Int.
Glucose (mmol/L)	6.45 ± 0.46	6.13 ± 0.49	7.15 ± 0.63 ^a^	5.93 ± 0.46 ^b^	****		**
Urea (mmol/L)	6.3 ± 0.6	6.1 ± 0.7	6.7 ± 0.7	7.3 ± 1.5		**	
Creatinine (μmol/L)	35.1 ± 4.1	32.9 ± 4.9	34.5 ± 6.7	40.3 ± 5.0 ^c^		*	*
Sodium (mmol/L)	141.2 ± 3.9	144.4 ± 1.8	147.9 ± 6.6 ^d^	146.3 ± 0.9		***	*
Potassium (mmol/L)	7.3 ± 0.5	7.1 ± 0.7	6.2 ± 0.7 ^e^	5.9 ± 0.5		****	

Abbreviations: C—control rats with standard diet, Q—rats with standard diet for 15 weeks and after administration of quercetin for the last 6 weeks of the experiment, F—rats fed by fructose for 15 weeks, FQ—rats fed by fructose for 15 weeks and after administration of quercetin for the last 6 weeks of the experiment, QUE—effect of quercetin administration, FRU—effect of fructose intake, Int.—interaction between the quercetin administration and fructose intake. Data represent mean ± SD. Statistical significance—a: *p* = 0.01 vs. C; b: *p* < 0.0001 vs. F; c: *p* < 0.05 vs. F; d: *p* < 0.005 vs. C; e: *p* < 0.0005 vs. C; * *p* < 0.05, ** *p* < 0.01, *** *p* < 0.001, **** *p* < 0.0001 for the corresponding factors and their interaction.

**Table 3:** Markers of antioxidant status and oxidative and carbonyl stress in the renal tissue.

	Experimental Groups				Two-Way ANOVA		
	C (n = 11)	Q (n = 12)	F (n = 12)	FQ (n = 12)	QUE	FRU	Int.
TBARS (μmol/L)	4.6 ± 1.6	5.4 ± 1.2	6.3 ± 1.3 ^a^	6.4 ± 1.2		***	
AOPP (μmol/g)	3.3 ± 0.5	2.5 ± 0.2 ^b^	3.0 ± 0.4	2.4 ± 0.2 ^c^	****		
AGEs (mg/g)	7.9 ± 1.1	8.5 ± 0.6	7.6 ± 0.9	8.1 ± 1.0	*		
FRUC (μmol/g)	32.1 ± 6.3	16.0 ± 2.0	41.1 ± 22.9	22.1 ± 7.6 ^d^	***		
TAC (mmol/L)	1.3 ± 0.3	1.2 ± 0.2	1.5 ± 0.2	1.4 ± 0.2		**	
FRAP (mmol/L)	1.06 ± 0.20	1.33 ± 0.24 ^e^	1.36 ± 0.18 ^f^	1.25 ± 0.12		*	**
GSH/GSSG	0.34 ± 0.04	0.31 ± 0.02 ^e^	0.30 ± 0.02 ^f^	0.28 ± 0.02	***	****	

Abbreviations: C—control rats with standard diet, Q—rats with standard diet for 15 weeks and after administration of quercetin for the last 6 weeks of the experiment, F—rats fed by fructose for 15 weeks, FQ—rats fed by fructose for 15 weeks and after administration of quercetin for the last 6 weeks of the experiment, QUE—effect of quercetin administration, FRU—effect of fructose intake, Int.—interaction between the quercetin administration and fructose intake. TBARS—thiobarbituric acid reactive substances, AOPP—advanced oxidation protein products, AGEs—advanced glycation end products, FRUC—fructosamine, TAC—total antioxidant capacity, FRAP—ferric reducing antioxidant power, GSH/GSSG—ratio of reduced to oxidized glutathione. Data represent means ± SD. Statistical significance—a: *p* < 0.05 vs. C; b: *p* < 0.0005 vs. C; c: *p* < 0.01 vs. F; d: *p* < 0.05 vs. F; e: *p* < 0.01 vs. C; f: *p* < 0.005 vs. C; * *p* < 0.05, ** *p* < 0.01, *** *p* < 0.001, **** *p* < 0.0001 for the corresponding factors and their interaction.

## Data Availability

The data that support the findings of this study are available in this article or from the corresponding author upon reasonable request.
